# Siple Dome ice reveals two modes of millennial CO_2_ change during the last ice age

**DOI:** 10.1038/ncomms4723

**Published:** 2014-04-29

**Authors:** Jinho Ahn, Edward J. Brook

**Affiliations:** 1 School of Earth and Environmental Sciences, Seoul National University, Seoul 151742, Korea; 2 College of Earth, Ocean and Atmospheric Sciences, Oregon State University, Corvallis, Oregon 97331, USA

## Abstract

Reconstruction of atmospheric CO_2_ during times of past abrupt climate change may help us better understand climate-carbon cycle feedbacks. Previous ice core studies reveal simultaneous increases in atmospheric CO_2_ and Antarctic temperature during times when Greenland and the northern hemisphere experienced very long, cold stadial conditions during the last ice age. Whether this relationship extends to all of the numerous stadial events in the Greenland ice core record has not been clear. Here we present a high-resolution record of atmospheric CO_2_ from the Siple Dome ice core, Antarctica for part of the last ice age. We find that CO_2_ does not significantly change during the short Greenlandic stadial events, implying that the climate system perturbation that produced the short stadials was not strong enough to substantially alter the carbon cycle.

## Abstract (web summary)

Whether all rapid climate events during the last ice age impacted the global carbon cycle is not clearly understood. Ahn and Brook present a high-resolution record of atmospheric CO_2_ from Antarctica and suggest that only Greenland stadials associated with massive iceberg discharge influenced atmospheric CO_2_.

## Graphical abstract

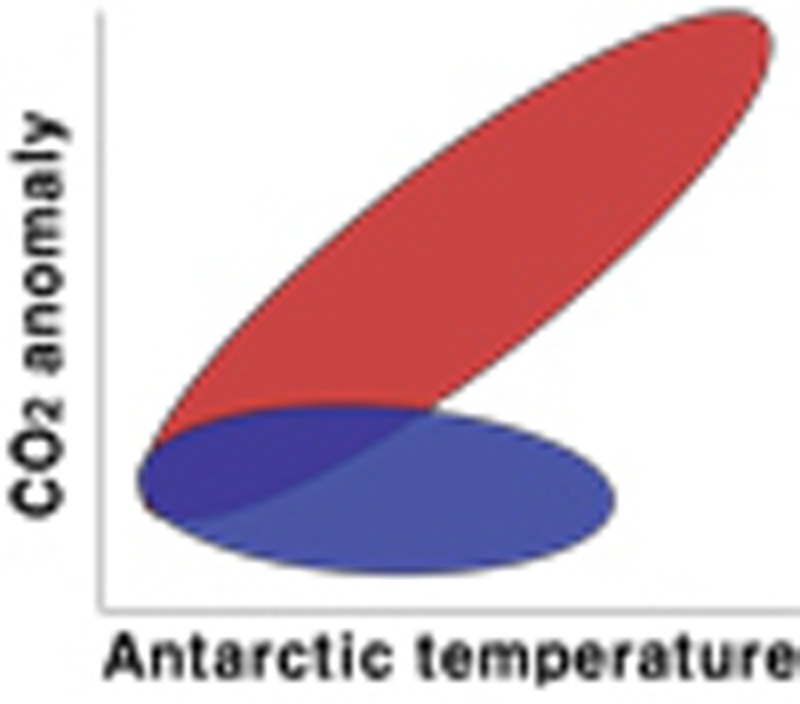

Ice core records from Greenland reveal a detailed history of abrupt climate change during the last glacial period. Warm and cold periods (interstadial and stadial, respectively) repeated on millennial timescales but rapid switches between the two happened in decades^1^,^2^,^3^,^4^. Antarctic ice core records, however, reveal gradual warming during Geenlandic stadials and cooling during interstadials^5^,^6^. The out-of-phase interhemispheric climate relationship is usually referred to as the ‘bipolar seesaw’^7^ and the most popular hypothesis for the control mechanism includes reorganization of ocean-atmosphere circulation and change in meridional heat transport, possibly caused by fresh water input into the North Atlantic^8^,^9^,^10^.

Reconstruction of atmospheric CO_2_ during abrupt climate change events may help us better understand climate-carbon cycle feedbacks and provide data for testing carbon cycle models under variety of boundary conditions. Existing Antarctic ice core records show CO_2_ increases during long Greenlandic stadials, which are also accompanied by major Antarctic warmings^11^,^12^. Ventilation of CO_2_-rich deep water in the Southern Ocean may have controlled ocean-atmosphere carbon exchange and therefore atmospheric CO_2_ concentration^13^. Marine sediment records from the Southern Ocean indicate increased opal flux^14^ and reduced stratification^15^ during the Younger Dryas event and the long stadial preceding the Bølling-Allerød event^14^,^15^, both times of rising CO_2_, and can be interpreted as a record of increased upwelling and CO_2_ outgassing in the Southern Ocean^14^,^15^. During the same time intervals, the Atlantic meridional overturning circulation (AMOC) was reduced and the Antarctic temperature gradually increased^16^,^17^. During the long stadials of the last ice age, marine sediment records indicate shoaled AMOC^18^ and increased opal flux^14^ in the Southern Ocean, although the chronology of the proxy for the latter is not well constrained. The shoaling of AMOC likely coincided with reduction in AMOC strength that might have caused reduction in northward oceanic heat transport, the gradual warming in Antarctica and CO_2_ outgassing from the Southern ocean during the long stadials^10^,^14^, analogous to the early stage of the last deglacial Antarctic warming and CO_2_ increase^14^,^19^,^20^.

High-resolution records from the EPICA Dronning Maud Land (EDML) ice core in east Antarctica show the ‘bipolar seesaw’ operated not only during the major long stadials but also during other short ones, and that stadial duration is positively correlated with the magnitude of the temperature increase in Antarctica. These observations support the hypothesis of reduction in AMOC during both long and short stadials^21^, although there is no clear marine evidence of AMOC reduction during each of the short stadials, perhaps owing to insufficient data resolution and/or chronology^6^.

By analogy to the relationship between CO_2_ and major Antarctic warmings/long Greenlandic stadials, we might expect small CO_2_ increases during the small Antarctic warmings/short Greenlandic stadials. However, atmospheric CO_2_ change during the short stadials is not well resolved in existing ice core records owing to low temporal data resolution (280–570 years^11^,^12^,^22^).

We investigate CO_2_ variations during the short Greenlandic stadials, with a multi-decadal to centennial CO_2_ record with a mean sampling resolution of 95 years, from the Siple Dome ice core, Antarctica. Our new results cover the time interval of Greenlandic abrupt climate events (Dansgaard-Oeschger or DO events) DO2–7 and our sampling resolution is sufficient to examine CO_2_ trends during the short stadials lasting for 800–1200 years. Combined with recently published high-resolution data for DO8–10 from the same core^23^, we constructed a complete high-resolution CO_2_ record from 22 to 41 ka.

## Results

### Natural smoothing of gas records in the Siple Dome ice core

**Figure 1:**
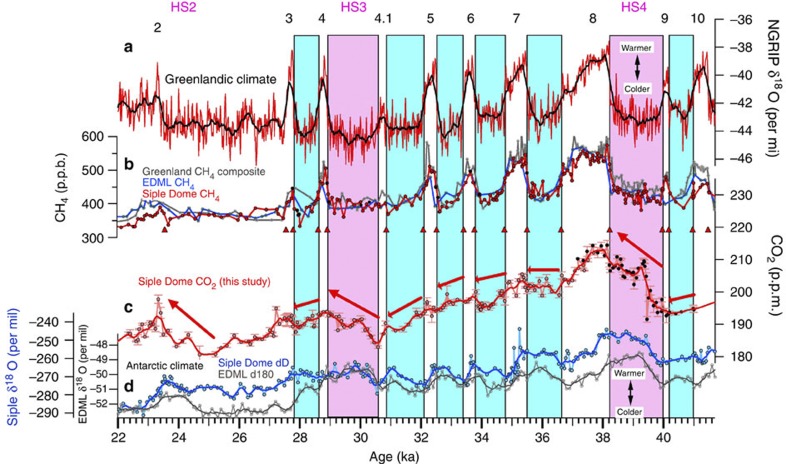
High-resolution CO_2_ and climate records during abrupt climate change during the last ice age. (**a**) Greenlandic isotopic temperature record from the NGRIP ice core^57^. Black numbers represent Dansgaard-Oeschger events. HS stands for Heinrich stadial, indicating long stadials that include Heinrich events. (**b**) Atmospheric CH_4_ records from Greenland (grey)^21^, Siple Dome (red)^24^ and EDML (blue)^21^ ice cores. Black dots are new Siple Dome CH_4_ data (this study). Red triangles indicate age control points. (**c**) Atmospheric CO_2_ record from Siple Dome, Antarctica ice core (this study). Red line represents 300-year running means of the CO_2_ record. Black dots are published records^23^. (**d**) Antarctic temperature proxy records from Siple Dome (dark blue)^24^ and EDML (grey)^21^ ice cores. All the ages are synchronized on Greenland Ice Core Chronology 2005 (GICC05) timescale. Blue and pink boxes indicate time intervals of short and long stadials (Greenlandic cold spans), respectively. During those stadials, Antarctic temperature increased.

Snow accumulation rates at the Siple Dome are relatively high, and therefore smoothing of gas records by diffusion and gradual bubble close-off in the firn (unconsolidated snow layer on the top of ice sheet) is relatively small^24^. The high snow accumulation rates also result in a small uncertainty in the relative timing between gas ages and ice ages (Δage)^25^, allowing better comparison of gas records with temperature proxy records^24^. A firn densification model for Siple Dome shows Δage of 500–1000 years during the 22–41-ka period^24^. Because the width of the gas age distribution at half-height is typically about 10% of Δage^25^,^26^,^27^,^28^, we estimate the gas age distribution of the Siple Dome record to be <100 years during the time interval of study. The sharp increases in the Siple Dome CH_4_ record (Fig. 1) clearly confirm that the smoothing of gas records is minimal on multi-centennial timescales and supports our estimation of smoothing of the Siple Dome CO_2_ record.

### Two modes of CO_2_ change during Greenlandic stadials

**Figure 2:**
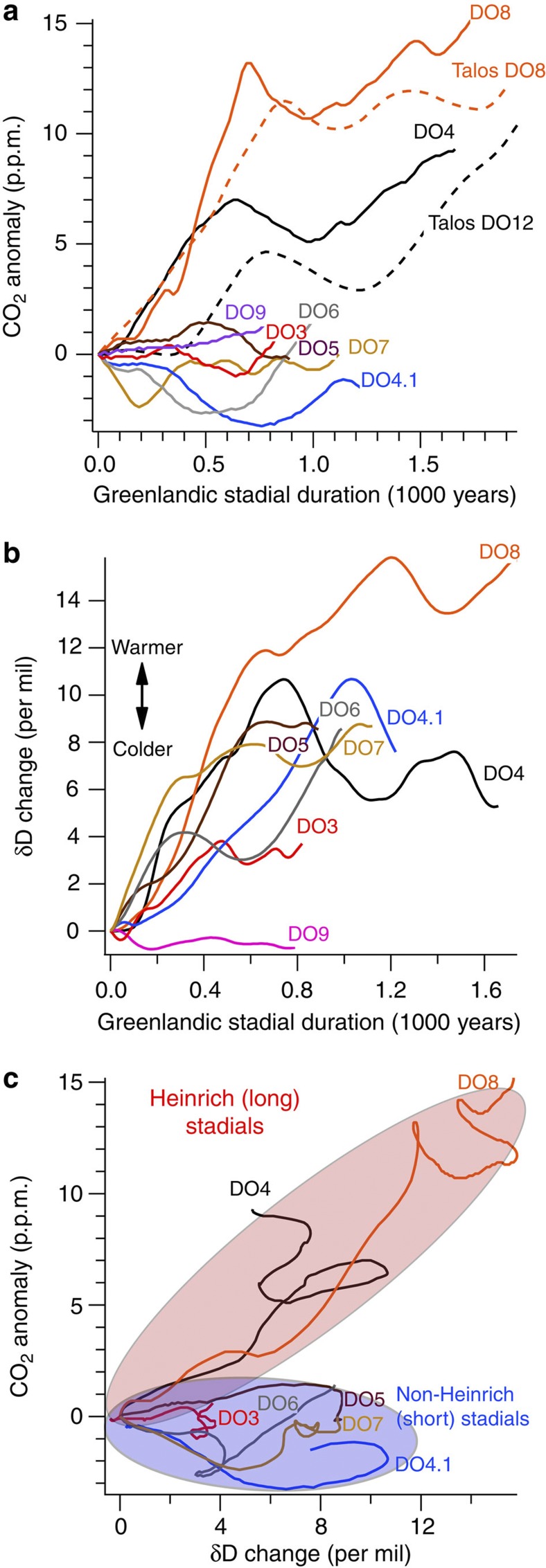
Time evolution of CO_2_ and Antarctic isotope temperature during Greenlandic stadials. (**a**) CO_2_ change during Greenlandic stadials from Siple Dome (solid lines) (this study) and Talos Dome (dashed lines)^12^ ice core records. DO numbers indicate DO warmings at the end of the stadials. (**b**) Antarctic temperature proxy record during stadials from Siple Dome ice core^24^. (**c**) Time evolution of atmospheric CO_2_ versus isotopic temperature anomalies during stadials. Derived from (**a**,**b**). The pale red and blue ellipses indicate records for Heinrich (long) and non-Heinrich (short) stadials, respectively. Three hundred-year running means are used for both CO_2_ and isotopic temperature proxy records. In order to remove multi-millennial changes during short Greenlandic stadials, the Siple Dome CO_2_ and isotopic temperature records are detrended.

As shown in Fig. 1, we observe small CO_2_ variations of ~5 p.p.m. on centennial timescales during the short stadials. A 300-year running mean (red curve) removes these features (Fig. 1), illustrating that CO_2_ change was negligible on multi-centennial timescales. We observe small decreases on longer timescales during most of the short stadials (Fig. 1), but these are part of a long-term trend. After detrending it becomes clear that the CO_2_ change associated with short stadials themselves is insignificant (Fig. 2a). In contrast, we observe CO_2_ increases during the long stadials, confirming previous results from different Antarctic ice cores^11^,^12^,^22^,^29^ (Fig. 2a). The isotopic temperature proxy (*δD*_ice_) from the Siple Dome ice core shows small Antarctic warmings during most of the short stadials (Fig. 2b) and confirms previous results from the EDML ice core^21^, implying that the small Antarctic warmings during the short stadials are not only local features but at least of larger regional extent because Siple Dome is located in the Pacific sector, while the EDML core is in the Atlantic sector in Antarctica. Combining the Siple Dome CO_2_ and climate records, we plot the time evolution of CO_2_ versus the temperature proxy (*δD*_ice_) anomalies during Greenlandic stadials or Antarctic warmings, using the detrended Siple Dome CO_2_ and temperature proxy records for short stadials (Fig. 2c). We find that CO_2_ and *δD*_ice_ anomalies are not significantly correlated during the short stadials (average *r*=0.0), but positively correlated during the long stadials (average *r*=0.84) (Fig. 2c). A slight temperature decrease in the Siple Dome *δD*_ice_ between DO9 and 10 is not confirmed in the EDML isotopic temperature (*δO*_ice_) record^21^ and excluded in our calculation. We note that Siple Dome isotopic temperature (*δD*_ice_) between DO3 and 4 increases, but at EDML it decreases, presumably owing to local effects. Our finding of the two different modes of CO_2_ change during Greenlandic stadials for the period 22–41 ka is consistent with the results of a recent, lower resolution study of CO_2_ variations from 38 to 115 ka^12^, which shows <5 p.p.b. variations in CO_2_ during the short stadial events of marine isotope stage 3.

## Discussion

**Figure 3:**
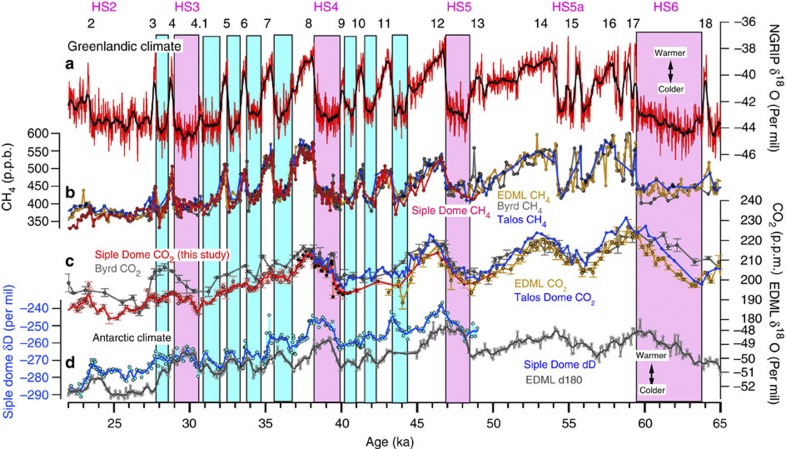
Atmospheric CO_2_ and climate records from multiple ice cores. (**a**–**d**) Ice core records extended from Fig. 1. Siple Dome CO_2_ and CH_4_ records are compared with existing low-resolution records from EPICA EDML^12^,^21^, Byrd^11^ and Talos^11^,^58^ ice cores, Antarctica. Age intervals for HS2 and HS5a are not well constrained owing to chronological uncertainty in the paleoproxy records.

The small-to-insignificant CO_2_ change during the short stadials may imply that AMOC perturbations happened at these events but were too short to result in a change in atmospheric CO_2_. If this were the case, we would expect to observe no CO_2_ increase during the first 800–1200 years of the long stadials, because duration of the short stadials ranges 800–1200 years. However, we observe that CO_2_ increases from the beginning of the long Greenlandic stadials predating DO8 and DO4 (Fig. 2a). The time lag of CO_2_ relative to the isotopic signal during Greenlandic stadials predating DO12 and 17 from other ice core records appears small as well^11^,^12^. However, we cannot clearly rule out the possibility of a time lag of several centuries (Fig. 3). In addition, there is some ambiguity about the start of the long stadial predating DO4, which is conventionally defined by the end of a small temperature proxy peak (DO4.1)^21^. We follow convention here, but note that additional high-resolution data from other long stadial events will be needed to further address the question of when CO_2_ starts to rise during events of this type. The above observation suggests that climate perturbations associated with the long and short stadials are different. Cave deposits reveal less weakening in the Asian monsoon^30^ and less intense South American monsoon^31^ during the short stadials compared with the long stadials, suggesting that the perturbation to the climate system related to the short stadial events in Greenland was weaker than for the long ones. A comparison of Antarctic ice core climate records with a thermodynamic model also indicates that the long stadials were caused by a stronger climate perturbation than short ones^32^. Finally, although it is not conclusive, *δ*^13^C in benthic foraminifera from North Atlantic sediment cores indicates less shoaling of AMOC during short stadials than that during the long stadials^33^. Thus it is likely that strength of the climate perturbation is related to change in atmospheric CO_2_ during the Greenlandic stadials. Massive iceberg discharge events in the North Atlantic (Heinrich events) occurred within time intervals of the long stadials. The Heinrich events could have increased fresh water forcing into the North Atlantic and also caused large perturbations to atmospheric circulation (for example, southward movement of the ITCZ^34^). However, multiple studies suggest that the Heinrich events lag onsets of long stadials^35^,^36^, although exact timing of those events within the stadials is not well constrained^37^,^38^.

The control mechanisms for the two CO_2_ modes may exist in oceanic processes such as AMOC reduction and consequent upwelling in the Southern Ocean. Those oceanic processes can be linked by change in vertical salinity transport and stratification in the Southern Ocean^39^ and/or latitudinal shift of Southern Hemisphere Westerlies^14^,^40^ and/or strength of the Southern Hemisphere Westerlies^41^,^42^,^43^. Although we cannot pinpoint a precise oceanic mechanism, we speculate that the weakening in AMOC during the short stadials might have not been sufficient to cause enough of a change in upwelling to impact atmospheric CO_2_. Marine proxy data for upwelling in the Southern Ocean do not clearly show strong peaks in between long stadials that bracket several short stadials^14^, supporting this hypothesis.

Other potential oceanic mechanisms that change CO_2_ outgassing include variations in sea ice extent and changes in iron fertilization in the Southern Ocean^44^. Sea-salt-Na may be a proxy for sea ice extent, but Siple Dome, Dome C and EDML ice core records do not show significant differences between long and short Greenlandic stadials^44^,^45^. Proxy records for the Fe-flux (non-sea-salt Ca) from Dome C and EDML cores show highly reduced Fe-flux during several long Greenlandic stadials that predate DO8, but after DO8 the reduction during long stadials is not larger than that during short stadials^44^. Thus a difference in iron fertilization in the Southern Ocean is not likely the main cause of the two modes in CO_2_ change.

Atmospheric CO_2_ can be also controlled by exchange of land carbon. Terrestrial carbon is mostly affected by temperature and precipitation because they both control vegetation and organic carbon in soil. Compared with interstadials, paleoproxy data for both short and long stadials indicate colder and dryer conditions in the northern hemisphere, and warmer and wetter conditions in the southern hemisphere, although the magnitude of those changes depends on the type of stadials^6^. However, model simulations predict either a decrease^46^,^47^ or increase^48^ in land carbon during the stadials. Although we cannot rule out terrestrial control on the two modes of CO_2_ change, we suggest that the control mechanism exists more likely in the ocean rather than on land, because we have supporting evidence for an oceanic CO_2_ source during the long stadials in the last deglacial period^14^,^15^,^49^,^50^.

In principle, the lack of change in atmospheric CO_2_ could also result from compensating changes in sources (for example, coincident terrestrial uptake and oceanic release) as predicted in models of AMOC shutdown and carbon cycle response^46^,^47^,^51^. However, the global impact of short stadials on the terrestrial biosphere was probably small, given that paleoproxy records indicate weaker terrestrial climate perturbations during the short Greenlandic stadials compared with the long ones^30^,^31^ as discussed above. Thus, terrestrial uptake balancing oceanic release during the short Greenlandic stadials is not likely the main explanation for the lack of CO_2_ response.

Our new high-resolution record defines two modes of millennial scale CO_2_ change during stadial events in the northern hemisphere that depend on the nature of the Greenlandic stadial. During short Greenlandic stadials, those not associated with Heinrich events, it is likely that the impact on ocean circulation was not sufficient to release CO_2_ from deep ocean to the atmosphere. The lack of correlation between CO_2_ and Antarctic temperature change during the short stadials implies that links between Antarctic climate change and high-latitude northern hemisphere climate may have been controlled by shallow oceanic and/or atmospheric processes, while CO_2_ change was controlled by deep oceanic and Southern Ocean processes.

## Methods

### CO_2_ concentration measurement

For CO_2_ analysis at the Oregon State University, samples were placed in a double-walled stainless steel chamber at −35 °C, cooled using cold ethanol circulation between the walls, evacuated for 13 min and then crushed with steel pins. Air liberated from the ice was dried in a cold stainless steel coil at −85 °C and then trapped in ~6 cm^3^ stainless steel sample tubes at −262 °C. After warming the trapped air to room temperature, the CO_2_ mixing ratio was measured with an Agilent 6890N Gas Chromatograph (GC) with a flame ionization detector, with nickel catalyst conversion of CO_2_ to CH_4_ before measurement. Daily calibration curves used several measurements of standard air with 197.54 p.p.m. CO_2_ (WMOX2007 CO_2_ mole fraction scale). Daily corrections for the dry extraction and GC analysis were done using several standard airs (197.54 p.p.m.) that were introduced over the ice samples and trapped in sample tubes mimicking the procedure of the air samples from ice. We compared 2–5 replicates from the same depths. Details of the methods are described in ref. 52. The s.e. for replicates from the same depth averaged 0.6 p.p.m. for the Siple Dome ice. The excellent agreement among the replicates were achieved by careful trimming of the ice surface and improved analytical techniques^52^ since our early analysis for the same core a decade ago at Scripps Institution of Oceanography^53^.

### CH_4_ concentration measurement

CH_4_ analysis was separately performed at the Oregon State University^54^. Duplicate samples with a weight of ~60 g for each were analysed for each depth interval. Samples were placed in cold glass flasks bathed in an ethanol bath at −64.5 °C. The flasks with the ice samples were evacuated for 1 h. The flasks valves were closed and then the ice was melted in a warm water bath. After melting, the flasks were submerged in the cold ethanol bath to refreeze the ice melt. Air liberated from each ice sample was analysed four times with an Agilent 6890N GC with a flame ionization detector. Data are reported on the NOAA04 methane concentration scale.

### Synchronization of ice core records

Our CO_2_ record from the Siple Dome core is synchronized with NGRIP (North Greenland Ice Core Project) ice ages on the Greenland Ice Core Chronology 2005 (GICC05) timescale^55^ using abrupt CH_4_ changes that are near synchronous with abrupt Greenlandic climate change^56^. We used updated CH_4_ records to make better synchronization. The CH_4_ data resolution is 82 and 232 years for 23.5–42.3 and 42.3–46.9 ka, respectively. The GICC05 timescale is based on layer counting of Greenland ice cores and agrees well with other absolute ages such as cave deposit records^55^. At DO2, the correlation between CH_4_ increase and Greenlandic warming is not clear, and thus we correlate Siple Dome CH_4_ with the NGRIP CH_4_ record. The age tie points are listed in Table 1 and their uncertainty is controlled primarily by the CH_4_ data resolution. The age differences were linearly interpolated at depths between the tie points, and we reconstructed new ages at those depths by adding the calculated difference to the original ages^24^. Synchronized ice ages were determined using published estimates of ice age-gas age difference^24^.

## Author contributions

J.A. designed and carried out the experiments. All authors interpreted the data and wrote the manuscript.

## Additional information

**How to cite this article:** Ahn, J. & Brook, E.J. Siple Dome ice reveals two modes of millennial CO_2_ change during the last ice age. *Nat. Commun.* 5:3723 doi: 10.1038/ncomms4723 (2014).

## Supplementary Material

Supplementary Data 1 Siple Dome ice core CO2 record

## Figures and Tables

**Table 1:** Gas age tie points for Siple Dome ice core.

**Event**	**Siple Dome depth (m)**	**GISP2 age (ka)24**	**GICC05 age (b2k)55**	**Age difference**
CH_4_ rise associated with				
DO2 onset	739.50	23.93	23.54	−0.39
DO3 end	757.90	27.61	27.54	−0.07
DO3 onset	760.62	28.02	27.78	−0.24
DO4 end	765.80	28.78	28.60	−0.18
DO4 onset	767.81	29.19	28.90	−0.29
DO4.1 onset	780.84	31.09	30.85	−0.24
DO5 end	784.70	31.65	32.07	0.42
DO5 onset	789.03	32.50	32.51	0.01
DO6 end	793.60	33.13	33.40	0.27
DO6 onset	795.03	33.73	33.76	0.03
DO7 end	803.70	34.59	34.75	0.16
DO7 onset	809.94	35.54	35.50	−0.04
DO8 end	816.00	36.31	36.62	0.31
DO8 onset	825.55	38.49	38.22	−0.27
DO9 end	837.40	40.01	39.96	−0.05
DO9 onset	838.32	40.12	40.17	0.05
DO10 end	841.00	40.47	40.96	0.49
DO10 onset	845.99	41.09	41.47	0.38
DO11 end	849.90	41.77	42.27	0.50
DO11 onset	855.28	42.82	43.42	0.60
DO12 end	860.10	43.85	44.34	0.49
DO12 onset	871.77	46.36	46.87	0.51
